# Associations Between Aldosterone-Renin-Ratio and Bone Parameters Derived from Peripheral Quantitative Computed Tomography and Impact Microindentation in Men

**DOI:** 10.1007/s00223-023-01131-x

**Published:** 2023-09-10

**Authors:** Kara L. Holloway-Kew, Kara B. Anderson, Pamela Rufus-Membere, Monica C. Tembo, Sophia X. Sui, Natalie K. Hyde, Mark A. Kotowicz, Stella M. Gwini, Jun Yang, Adolfo Diez-Perez, Maciej Henneberg, Wan-Hui Liao, Julie A. Pasco

**Affiliations:** 1 IMPACT – the Institute for Mental and Physical Health and Clinical Translation, School of Medicine, Deakin University, Geelong, Australia; 2 Barwon Health, Geelong, Australia; 3 Department of Medicine - Western Health, The University of Melbourne, St Albans, Australia; 4 Centre for Endocrinology and Metabolism, Hudson Institute of Medical Research, Clayton, Australia; 5 School of Public Health and Preventive Medicine, Monash University, Prahran, Australia; 6 Department of Medicine, Monash University, Clayton, Australia; 7 Barcelona- Department of Internal Medicine, Hospital del Mar-IMIM, Instituto Carlos III, Autonomous University of Barcelona and CIBERFES, Barcelona, Spain; 8 Biological Anthropology and Comparative Anatomy Research Unit, School of Biomedicine, University of Adelaide, Adelaide, Australia; 9 Institute of Evolutionary Medicine, University of Zurich, Zurich, Switzerland; 10 Department of Internal Medicine, Taipei City Hospital Yangming Branch, Taipei City, Taiwan

**Keywords:** Aldosterone-renin-ratio, Primary aldosteronism, Impact microindentation, Bone material strength index, Peripheral quantitative computed tomography, Males

## Abstract

Components of the renin–angiotensin–aldosterone system (RAAS) are present on bone cells. One measure of RAAS activity, the aldosterone-renin-ratio (ARR), is used to screen for primary aldosteronism. Associations between ARR and bone mineral density are conflicting. This study investigated associations between ARR and peripheral quantitative computed tomography (pQCT) and impact microindentation (IMI). Male participants (n = 431) were from the Geelong Osteoporosis Study. “Likely” primary aldosteronism was defined as ARR ≥ 70 pmol/mIU. Another group, “possible” primary aldosteronism, was defined as either ARR ≥ 70 pmol/mIU or taking a medication that affects the RAAS, but not a beta blocker, and renin < 15 mU/L. Using pQCT, images at 4% and 66% of radial (n = 365) and tibial (n = 356) length were obtained. Using IMI measurements, bone material strength index (BMSi; n = 332) was determined. Associations between ARR or likely/possible primary aldosteronism and IMI or pQCT-derived bone parameters were tested using median regression. ARR and aldosterone values were not associated with any of the pQCT-derived bone variables in either unadjusted or adjusted analyses. Men with likely primary aldosteronism (n = 16), had lower adjusted total bone area (radial 66% site, − 12.5%). No associations were observed for men with possible primary aldosteronism (unadjusted or adjusted). No associations with BMSi were observed (p > 0.05). There were no associations between ARR or aldosterone and pQCT-derived bone parameters. Men with likely primary aldosteronism had lower bone area, suggesting clinically high levels of ARR may have a negative impact on bone health.

**Electronic supplementary material:**

The online version of this article (doi:10.1007/s00223-023-01131-x) contains supplementary material, which is available to authorized users.

## Introduction

The renin–angiotensin–aldosterone system (RAAS) is responsible for maintaining blood pressure as well as fluid and electrolyte balance. Aldosterone activates the mineralocorticoid receptors in the renal tubules to increase sodium reabsorption, which leads to an increase in blood pressure [1]. Excess aldosterone occurs in primary or secondary hyperaldosteronism. Overproduction of aldosterone in the latter is triggered by its physiologic regulatory factors: renin, angiotensin II, corticotropin and potassium. Conversely, primary aldosteronism is caused by autonomous and unregulated aldosterone production by adrenal glomerulosa cells and is a common but under-diagnosed form of secondary hypertension [2]. Screening for primary aldosteronism is performed by measuring plasma levels of two components of the RAAS; aldosterone and renin, and calculating the aldosterone-to-renin ratio (ARR). The ARR value is important for differentiating between primary and secondary aldosteronism. In primary aldosteronism, excess aldosterone results in negative feedback and suppressed renin concentration, whereas in secondary aldosteronism, excess activation of the RAAS results in higher renin and a concomitant increase in aldosterone. Although the threshold for abnormal ARR varies, it is generally agreed that values ≥ 70 (pmol/mIU) indicate that primary aldosteronism may be present [3, 4].

Elements of the RAAS are present in bone cells, hence, dysregulation of aldosterone may impact bone health. In particular, mineralocorticoid receptors are present in bone tissue [5] and could affect bone through several possible mechanisms [6, 7]. Osteoblasts and osteocytes express 11β-hydroxysteroid dehydrogenase-2, an enzyme that inactivates cortisol and allows aldosterone to function [8]. Circulating cortisol concentrations are 1000 times higher than circulating aldosterone and in tissues that do not express 11β-hydroxysteroid dehydrogenase-2, mineralocorticoid receptors bind almost exclusively to cortisol. For bone cells that do express 11β-hydroxysteroid dehydrogenase-2, cortisol is inactivated, allowing aldosterone to bind to the mineralocorticoid receptors. Therefore, aldosterone may directly affect the differentiation, proliferation and function of osteoclasts, osteoblasts and osteocytes through mineralocorticoid receptors present on these cells [5]. Another possible mechanism is by increasing parathyroid hormone concentrations through binding to mineralocorticoid receptors located in the parathyroid glands or indirectly through increasing urinary calcium excretion [6, 9]. Both mechanisms result in a negative calcium balance, leading to a potential decrease in calcium in bone. Aldosterone may also affect bone by increasing oxidative stress, which can lead to increased osteoblast and osteocyte apoptosis [6]. Additionally, previous studies have reported that individuals with primary aldosteronism have lower vitamin D concentrations [7, 10], which is important for calcium and phosphorus homeostasis [11]. Currently the reason for this observation is not clear [10].

Few studies have investigated associations between aldosterone, renin or ARR and bone health. Two studies reported that although risk of fracture was higher in patients with primary aldosteronism, the differences in bone mineral density (BMD) were conflicting [9, 12]. Another study has demonstrated that trabecular bone score, which reflects bone microarchitecture [13], was lower for women, but not men, with primary aldosteronism compared to those without the condition, which may assist in explaining the increased risk of fracture [14]. The study also reported no differences in BMD for men or women. These studies suggest that other components of bone strength may be affected by changes in ARR, such as bone quality, rather than bone mass.

Alternative measures of bone that could be useful for investigating the effect of aldosterone, renin or ARR include peripheral quantitative computed tomography (pQCT) and impact microindentation (IMI). The pQCT technique provides information about bone microarchitecture at the radius and tibia [15]. It can differentiate between cortical and trabecular bone and provides important three-dimensional (volumetric) rather than two-dimensional (areal) BMD values. To our knowledge, only one study on this topic has used pQCT [16], showing that renin and ARR were associated with greater trabecular density, but no associations with aldosterone were observed. IMI is a technique that measures the fracture resistance of cortical bone at the mid-tibia using a device known as the OsteoProbe [17]. The device works by measuring the microindentation distance of the device tip and comparing it to the indentation distance in a polymethyl methacrylate control material [17, 18]. The ratio of these distances is calculated and expressed as a unitless variable known as the bone material strength index (BMSi). A bone that is more resistant to microcracks will have a smaller indentation depth relative to the control and will have a higher BMSi. We are not aware of any studies that have used IMI to study the effect of varying ARR values on bone.

Previous literature has indicated that individuals with primary aldosteronism may have deficits in bone quality rather than bone mass [14], and this may be better captured by these alternative measures of bone health. Therefore, the aim of this study was to investigate associations between a marker of primary aldosteronism, the ARR, and bone measures derived from pQCT and IMI techniques in a population-based sample of men.

## Methods

### Participants

Participants for this study were drawn from the Geelong Osteoporosis Study, a longitudinal cohort study situated in south-eastern Australia [19]. Participants were randomly recruited from the Australian electoral roll using an age-stratified sampling method. This study used data from the most recent assessment phase for men, the 15-year follow-up (data collected between 2016 and 2022). This was the first visit where pQCT and IMI techniques were performed. There were 448 men who provided blood samples at the 15-year follow-up and had data for pQCT and/or IMI.

### Biochemical Data

Blood samples were collected in the morning following an overnight fast. Aldosterone (pmol/L) and renin (mIU/L) were measured in plasma and the aldosterone-renin-ratio (ARR; pmol/mIU) was calculated. Plasma aldosterone concentration (PAC) and direct renin concentration (DRC) measurements were performed using the DiaSorin LIAISON® chemiluminescent immunoassays on the Liaison XL analyser (DiaSorin, Saluggia, Italy). PAC by the LIAISON® aldosterone assay was reported in pmol/L; the quoted between-run analytical coefficients of variation (CV) were 9.5% at 188 pmol/L and 5.6% at 798 pmol/L. The DRC was reported in mU/L; the quoted between-run analytical CV were 10.0% at 5.1 mU/L and 4.3% at 82.4 mU/L.

Other biochemical measurements were: estimated glomerular filtration rate (eGFR), serum calcium, parathyroid hormone, 25-hydroxy vitamin D (25OHD), sodium, potassium and bone turnover markers; C-terminal telopeptide (CTx) and procollagen type 1 N propeptide (P1NP).

### Peripheral Quantitative Computed Tomography (pQCT) Measurements

A peripheral computed tomography instrument (XCT 2000, Stratec Medizintechnik, Pforzheim, Germany) was used to obtain standard transverse scans at 4% and 66% of radial and tibial length from the distal end of the bone. The standard software (BonAlyse Oy, Jyvaskyla, Finland) was then used to analyse the scans and determine bone parameters. The quality of all scans was assessed using published protocols [20, 21] by at least two of the authors. The *in-vivo* CV for pQCT variables on this scanner were in the range of 0.9–3.9% for radial measures and 0.3–3.4% for tibial measures.

The pQCT-derived bone variables included in this study for the 4% site included Bone Mineral Content (g/cm), Total Area (mm^2^), Total Density (mg/cm^3^), Trabecular Area (mm^2^), Trabecular Density (mg/cm^3^), Cortical Area (mm^2^) and Cortical Density (mg/cm^3^). For the 66% site: Bone Mineral Content (g/cm), Total Area (mm^2^), Total Density (mg/cm^3^), Cortical Area (mm^2^), Cortical Density (mg/cm^3^), Cortical Thickness (mm) and Polar Stress Strain Index (mm^3^). Polar Stress Strain Index provides data regarding bone bending and torsional strength.

### Impact Microindentation (IMI)

An OsteoProbe device (Active Life Technologies, Santa Barbara, CA, USA) was used to perform IMI measurements at the mid-tibia and determine BMSi. The location of the measurements was determined by measuring the midpoint from the medial border of the tibial plateau to the distal edge of the medial malleolus. The measurement area was then disinfected and a local anaesthesia was applied. The probe tip was inserted through the skin to rest on the bone surface. The operator then pressed down on the outer housing of the device to perform the measurements. These measurements were conducted using the international recommended guidelines [22]. As we have reported previously [23], participants experienced minimal discomfort during measurements.

The first measurement was systematically discarded for each participant as it is commonly affected by insufficient penetration through the periosteum. Then at least ten measurements were performed, with the probe tip being moved approximately 2 mm between each one. At the time of data collection, there was no automated system for removal of invalid measurements and therefore we followed the recommended guidelines [22]. Measurements were removed if they appeared outside the “green zone” area indicated by the software, or if the operator reported abnormal bone “texture” during the measurements. There were three trained operators performing IMI measurements during the relevant follow-up phase, however, the majority (90.7%) were performed by a single operator (PR-M). The *in-vivo* CV for microindentation was 2% for repeated measures. This was calculated by performing the IMI measurement twice for 10 different participants. For each participant, the CV was calculated, showing precision between the two measurements performed. The final CV was expressed as the average of (%) SD/mean for all participants.

### Other Data

Weight was measured using electronic scales to the nearest 0.1 kg. Height was measured using a Harpenden stadiometer to the nearest 0.001 m. Body mass index (BMI) was calculated as weight (kg)/height(m)^2^. Systolic and diastolic blood pressure (mmHg) were measured using an automated device (Takeda Medical UA-751) while the participant was in a seated position. Hypertension was defined as systolic blood pressure ≥ 140 mmHg and/or diastolic blood pressure ≥ 90 mmHg.

The following data were obtained by self-report: Mobility data were collected as previously described [19], using a seven-point scale which included very active, active, sedentary, limited, inactive, chair or bedridden and bedfast. These categories were dichotomised into “high” mobility (very active and active) and “low” mobility (all other categories). Smoking status was categorised as currently smoking or not. Alcohol consumption was collected using a Food Frequency Questionnaire, developed by the Victorian Cancer Council [24]. This was dichotomised into “low” or “high” consumption; < 30 g or ≥ 30 g of alcohol per day, respectively. Prior low trauma fractures were determined by self-report and confirmed using radiological reports where possible, with 71.4% of fractures able to be confirmed. Fractures of the skull, face and digits were excluded. Medication use was self-reported and included angiotensin-converting enzyme inhibitors (ACEI), angiotensin II receptor blockers (ARB), diuretics, dihydropyridine calcium channel blockers (amlodipine, lercanipidine and felodipine), beta blockers and calcium supplements. Men taking oral glucocorticoids (n = 9) or anti-fracture medications (n = 8) were excluded from this study, leaving a total sample size of n = 431. One participant taking spironolactone was included in this study.

A combination of self-reported, measured and linkage data was used to calculate the Charlson comorbidity index (CCI) [25]. Non self-reported data included diabetes status, which was classified as fasting plasma glucose ≥ 7.0 mmol/L (126 mg/dL), self-reported diabetes and/or use of antihyperglycemic medications. Data linkage with the Victorian Cancer Registry provided all data on cancers from 1986 onwards. Rheumatoid arthritis (considered under connective tissue disease) was self-reported and confirmed using medication and medical record data from the major public hospital in the study region, the University Hospital Geelong. Acquired immunodeficiency syndrome was ascertained first through examination of self-reported medications, followed by University Hospital Geelong records. The remaining data for the CCI data were obtained via self-report.

Participant address details at a street level were used to ascertain socioeconomic status using the Index of Relative Socio-Economic Advantage and Disadvantage (IRSAD), which accounts for both disadvantage and advantage and includes income and occupation [26]. The IRSAD scores were divided into quintiles, where quintile 1 represented the most disadvantaged and quintile five the most advantaged.

Study data were collected and managed using REDCap electronic data capture tools hosted at Barwon Health [27, 28]. REDCap (Research Electronic Data Capture) is a secure, web-based software platform designed to support data capture for research studies, providing (1) an intuitive interface for validated data capture; (2) audit trails for tracking data manipulation and export procedures; (3) automated export procedures for seamless data downloads to common statistical packages; and (4) procedures for data integration and interoperability with external sources.

### Statistical Analysis

A total of 431 men were included in this study. The Shapiro–Wilk test, examination of box-plots and histograms were used to determine the normality for continuous variables. Weight, height, systolic and diastolic blood pressure and serum calcium were normally distributed and hence were described using means and standard deviations (SD). The remaining continuous variables were described using medians with 25th and 75th percentile (P25 and P75 respectively). Categorical variables were described using frequency and percentage. Differences between groups (those with and without likely primary aldosteronism as well as those with and without possible primary aldosteronism) were determined using t-tests for normally distributed continuous variables, Mann–Whitney tests for skewed continuous variables and chi-squared test for categorical variables.

Scatterplots of bone measures and ARR indicated a possible non-linear association, hence ARR values were natural log transformed prior to analyses. Associations between ARR and bone parameters (derived from pQCT or IMI) were assessed using median regression. Both unadjusted and adjusted estimates were reported together with their 95% confidence intervals (CI). The adjusted multivariable median regression models included the following likely confounders: age, weight, height, mobility, smoking status, alcohol consumption, prior fracture, CCI and medications affecting blood pressure (ACEI, ARB, diuretics and dihydropyridine calcium channel blockers). Since weight and height are likely to be correlated, we also performed the analyses using BMI and height instead. Other variables in this study were not included in the models as they were likely to be involved in the causal pathway for the association between ARR and bone parameters (e.g. hypertension and biochemical data). Additionally, due to the small number of men with high ARR values, we avoided including an excessive number of confounders in the models. Analyses were also performed excluding prior fracture to examine if including this variable resulted in over-adjustment in the models.

Additional analyses were conducted comparing bone parameters for men with likely or possible primary aldosteronism, as described in criteria used by Chee et al. [29]. An abnormal screening test for primary aldosteronism (henceforth referred to as “likely” primary aldosteronism), was defined as ARR ≥ 70 pmol/mIU [3], versus those without. Another analysis was also completed which included men categorised as having (i) “likely” primary aldosteronism (ARR ≥ 70 pmol/mIU) or ii) “possible” primary aldosteronism, defined as the presence of low renin concentration (< 15mIU/L) while taking RAAS-active medications that are known to increase renin concentration (ACEIs, ARBs, diuretics, dihydropyridine calcium channel blockers) but not taking a beta blocker, as these tend to reduce renin. These two groups of men [(i) and (ii)] were combined (henceforth referred to as “possible” primary aldosteronism) and compared to those who did not meet either criteria. Median (P25, P75) values for bone parameters were presented and differences between groups were identified using median regression analyses as described above.

An analysis was also completed to examine the association between aldosterone concentration (as a continuous variable) and bone parameters using the same methods as described above.

Bonferroni correction for multiple comparisons was applied, which adjusted the pre-specified level of statistical significance (alpha = 0.05) to alpha = 0.007. Analyses were completed using Minitab (Minitab, version 19, State College, PA, USA) and Stata (Version 17. StataCorp. 2017. Stata Statistical Software: Release 17. College Station, TX: StataCorp LLC).

## Results

### Descriptive Statistics

A description of participants is shown in Table 1. The median age was 64.7 years, with a range of 33 to 92 years. Overall, participants were in the overweight category for BMI (mean 27.7 kg/m^2^). Few men were current smokers, and few used calcium supplements (both < 10%). Approximately one in five were “high” consumers of alcohol, one in nine had suffered a previous low trauma fracture and one in ten (9.5%) were taking a beta blocker. Of the whole cohort, 16 (3.7%) had ARR ≥ 70 pmol/mIU.

**Table 1:** Descriptive characteristics of the participants included in this study (n = 431)

Age (years) (median P25, P75)	64.7 (54.2–73.6)
Weight (kg) (mean ± SD)	84.5 ± 13.6
Height (cm) (mean ± SD)	174.5 ± 6.9
Body mass index (kg/m^2^) (mean ± SD)	27.7 ± 3.9
Systolic blood pressure (mmHg) (mean ± SD)	140.6 ± 16.6
Diastolic blood pressure (mmHg) (mean ± SD)	79.2 ± 9.8
Hypertension (n %)	48 (11.1)
Low mobility (n %)	100 (23.2)
Current smoker (n %)	29 (6.7)
High alcohol consumption^a^ (n %)	90 (20.9)
Prior fracture (n %)	49 (11.4)
Charlson comorbidity index (median P25, P75)	0 (0–1)
Socioeconomic status^b^ (n %)	
Quintile 1 (most disadvantaged)	71 (16.5)
Quintile 2	80 (18.6)
Quintile 3	101 (23.4)
Quintile 4	112 (26.0)
Quintile 5 (most advantaged)	60 (13.9)
Biochemical data	
Aldosterone (pmol/L) (median P25, P75)	279.0 (200.0–387.0)
Renin (mIU/L) (median P25, P75)	24.5 (13.8–56.6)
Aldosterone-Renin Ratio (pmol/mIU) (median P25, P75)	11.4 (5.3–20.4)
Estimated glomerular filtration rate (mL/min/1.73m^2^) (median P25, P75)	74.5 (65.0–85.0)
Serum calcium (mmol/L) (mean ± SD)	2.25 ± 0.09
Parathyroid hormone (pmol/L) (median P25, P75)	5.4 (4.3–7.0)
Vitamin D (nmol/L) (median P25, P75)	64.0 (47.3–78.0)
Sodium (mmol/L) (median P25, P75)	140 (139–142)
Potassium (mmol/L) (median P25, P75)	4.2 (4.1–4.4)
C-terminal telopeptide (CTx, ng/L) (median P25, P75)	361 (272–467)
Procollagen type 1 N propeptide (P1NP, mcg/L) (median P25, P75)	47 (37–59)
Medication use	
Angiotensin-converting enzyme inhibitors (n %)	74 (17.2)
Angiotensin II receptor blockers (n %)	75 (17.4)
Diuretics (n %)	44 (10.2)
Dihydropyridine calcium channel blockers (n %)	52 (12.1)
Calcium supplements (n %)	20 (4.6)
Beta blockers (n %)	41 (9.5)

Data presented as mean ± SD, median (P25, P75) or n (%), as appropriate. Missing data: IRSAD n = 7, estimated glomerular filtration rate n = 3, calcium level n = 3, parathyroid hormone n = 11, vitamin D level n = 7, sodium level n = 3, potassium level n = 3, CTx n = 4, P1NP n = 4

^a^High consumption defined as ≥ 30 g alcohol per day

^b^Index of Relative Socio-economic Advantage and Disadvantage

Some men (38.8%) were taking at least one medication that affects the RAAS system (ACEI, ARB, diuretic and/or dihydropyridine calcium channel blocker). Of these men, 19.2% were also taking a beta blocker. Among men taking a medication that affects the RAAS system and not taking a beta blocker (n = 135), two (1.5%) had ARR ≥ 70 pmol/mIU and 18 (13.3%) had renin < 15 mIU/L. Additionally, there were 48 men with hypertension, and of these, six (12.5%) had ARR ≥ 70 pmol/mIU.

Men with likely primary aldosteronism (ARR ≥ 70 pmol/mIU) were taller (mean ± SD; 177.2 ± 4.8 vs 174.4 ± 6.9 cm, p = 0.035), more likely to have hypertension (37.5% vs 10.1%, p < 0.001) and had lower vitamin D (median P25, P75: 52.0; 37.0–65.8 vs 64.0; 48.0–78.0 nmol/L, p = 0.032) compared to men without likely primary aldosteronism (Supplementary Table 1).

Men with possible primary aldosteronism were more likely to have hypertension (21.9% vs 10.3%, p = 0.045) and higher systolic blood pressure (mean ± SD: 148.8 ± 16.6 vs 139.9 ± 16.4 mmHg, p = 0.006) than men who did not meet the criteria for possible primary aldosteronism (Supplementary Table 2). These men were also older (median P25, P75: 69.1; 61.1–81.9 vs 64.4; 53.7–73.5 years, p = 0.014), had higher sodium blood concentration (median P25, P75: 141; 140–143 vs 140; 139–141 mmol/L, p = 0.016), higher CTx (median P25, P75: 426; 338–626 vs 357; 269–459 ng/L, p = 0.007) and higher P1NP (median P25, P75: 54; 44–67 vs 46; 37–58 mcg/L, p = 0.032). There was also some evidence that these men had higher parathyroid hormone concentration (median P25, P75: 6.2; 4.9–7.9 vs 5.4; 4.3–6.9 pmol/L, p = 0.073) and lower vitamin D (median P25, P75: 53.5; 41.0–71.5 vs 64.0; 48.0–78.0 nmol/L, p = 0.057), but these did not reach statistical significance.

### Peripheral Quantitative Computed Tomography (pQCT)

For pQCT measurements, 397 men had a radial scan. Of these, 32 were excluded due to movement (n = 3) or measurement error (e.g. inability to determine the correct reference location, n = 29). There were 386 men who completed a tibial scan. Of these, 30 were excluded due to measurement error. This left 365 radius and 356 tibia scans available for analysis.

ARR as a continuous variable was not associated with any of the bone parameters derived from pQCT in either unadjusted or adjusted analyses. However, there was a trend towards lower adjusted bone mineral content at the tibia 4% site (Table 2).

**Table 2:** Associations between aldosterone-renin-ratio (ARR, pmol/mIU, natural log transformed) and peripheral quantitative computed tomography derived bone parameters

	Summary of bone parameters	Results from median regression for the association between ARR (pmol/mIU) and bone parameters			
	Median (P25, P75)	Unadjusted		Adjusted^*^	
		β coefficient (95%CI)	p value	β coefficient (95%CI)	p value
Radius 4% site					
Bone Mineral Content (g/cm)	1.640 (1.480, 1.790)	− 0.006 (− 0.032, 0.020)	0.663	0.009 (− 0.017, 0.035)	0.497
Bone Total Area (mm^2^)	508.0 (454.9, 558.1)	2.733 (− 6.253, 11.720)	0.550	3.339 (− 4.325, 11.004)	0.392
Bone Total Density (mg/cm^3^)	330.3 (295.9, 359.0)	− 1.386 (− 6.381, 3.610)	0.586	− 1.426 (− 6.192, 3.340)	0.557
Bone Trabecular Area (mm^2^)	228.5 (204.6, 251.0)	1.250 (− 2.772, 5.272)	0.542	1.508 (− 1.950, 4.965)	0.392
Bone Trabecular Density (mg/cm^3^)	217.2 (188.5, 242.3)	0.446 (− 3.977, 4.870)	0.843	3.449 (− 0.576, 7.475)	0.093
Bone Cortical Area (mm^2^)	279.5 (250.3, 307.1)	1.484 (− 3.480, 6.448)	0.557	1.832 (− 2.359, 6.022)	0.391
Bone Cortical Density (mg/cm^3^)	419.4 (374.3, 460.8)	− 5.060 (− 11.900, 1.780)	0.147	− 3.674 (− 9.715, 2.366)	0.232
Radius 66% site					
Bone Mineral Content (g/cm)	1.395 (1.270, 1.510)	− 0.004 (− 0.026, 0.018)	0.702	0.000 (− 0.020, 0.020)	0.994
Bone Total Area (mm^2^)	174.9 (160.3, 194.1)	− 1.564 (− 4.276, 1.149)	0.258	− 2.141 (− 4.542, 0.260)	0.080
Bone Total Density (mg/cm^3^)	805.5 (733.2, 858.4)	5.817 (− 4.845, 16.479)	0.284	7.115 (− 2.845, 17.075)	0.161
Bone Cortical Area (mm^2^)	108.0 (96.6, 116.8)	− 1.051 (− 2.738, 0.635)	0.221	− 0.323 (− 1.844, 1.199)	0.677
Bone Cortical Density (mg/cm^3^)	1147.5 (1119.8, 1168.4)	1.867 (− 2.341, 6.075)	0.383	0.354 (− 2.851, 3.558)	0.828
Bone Cortical Thickness (mm)	2.850 (2.571, 3.081)	0.020 (− 0.021, 0.060)	0.339	0.008 (− 0.029, 0.046)	0.656
Polar Stress Strain Index (mm^3^)	416.1 (361.9, 473.0)	0.183 (− 9.028, 9.395)	0.969	− 4.514 (− 12.407, 3.379)	0.261
Tibia 4% site					
Bone Mineral Content (g/cm)	4.260 (3.840, 4.640)	− 0.047 (− 0.116, 0.022)	0.177	− 0.078 (− 0.142, − 0.014)	0.017
Bone Total Area (mm^2^)	1325.3 (1220.8, 1431.8)	− 3.896 (− 21.524, 13.732)	0.664	− 3.545 (− 17.311, 10.221)	0.613
Bone Total Density (mg/cm^3^)	320.1 (298.4, 350.3)	− 0.355 (− 4.741, 4.032)	0.874	− 1.724 (− 5.506, 2.058)	0.371
Bone Trabecular Area (mm^2^)	596.3 (549.3, 644.3)	− 1.759 (− 9.691, 6.173)	0.663	− 1.567 (− 7.766, 4.632)	0.619
Bone Trabecular Density (mg/cm^3^)	254.0 (229.0, 276.1)	− 1.651 (− 6.031, 2.728)	0.459	− 1.958 (− 6.247, 2.331)	0.370
Bone Cortical Area (mm^2^)	729.0 (671.5, 787.5)	− 2.136 (− 11.833, 7.560)	0.665	− 1.978 (− 9.545, 5.589)	0.608
Bone Cortical Density (mg/cm^3^)	376.6 (349.1, 411.1)	− 2.009 (− 7.096, 3.079)	0.438	− 3.510 (− 8.108, 1.087)	0.134
Tibia 66% site					
Bone Mineral Content (g/cm)	4.690 (4.285, 5.080)	0.000 (− 0.070, 0.070)	> 0.999	− 0.030 (− 0.085, 0.025)	0.289
Bone Total Area (mm^2^)	754.5 (694.4, 821.3)	− 0.505 (− 11.568, 10.558)	0.928	0.334 (− 10.375, 11.043)	0.951
Bone Total Density (mg/cm^3^)	624.8 (571.2, 678.6)	− 6.710 (− 15.853, 2.433)	0.150	− 9.081 (− 18.448, 0.286)	0.057
Bone Cortical Area (mm^2^)	355.8 (319.0, 385.4)	− 0.313 (− 5.849, 5.223)	0.912	− 1.053 (− 5.573, 3.467)	0.647
Bone Cortical Density (mg/cm^3^)	1112.7 (1087.9, 1133.6)	0.226 (− 3.776, 4.227)	0.912	− 0.181 (− 3.845, 3.483)	0.923
Bone Cortical Thickness (mm)	4.266 (3.800, 4.687)	− 0.043 (− 0.119, 0.033)	0.269	− 0.059 (− 0.133, 0.015)	0.121
Polar Stress Strain Index (mm^3^)	3292.7 (2930.5, 3629.3)	2.668 (− 60.053, 65.388)	0.933	− 21.034 (− 61.431, 19.363)	0.306

*Other variables tested in the models: age, weight, height, mobility, smoking status, alcohol consumption, prior fracture, Charlson comorbidity index, angiotensin-converting enzyme inhibitors, angiotensin II receptor blockers, diuretics and dihydropyridine calcium channel blockers

Men with likely primary aldosteronism (ARR ≥ 70 pmol/mIU, n = 16) had lower adjusted total area at the 66% radial site (− 12.5% compared to median for those without primary aldosteronism, Table 3). There were also trends towards lower adjusted bone mineral content at the 4% radial and tibial sites as well as the 66% radial site.

**Table 3:** Relationship between peripheral quantitative computed tomography derived bone parameters and likely primary aldosteronism status (Aldosterone-renin-ratio (ARR) ≥ 70 pmol/mIU)

	Summary of bone measure by ARR category		Comparison of bone measures between the two categories (reference = ARR < 70 pmol/mIU)			
	ARR < 70 pmol/mIU (n = 415)	ARR ≥ 70 pmol/mIU (n = 16)	Unadjusted		Adjusted*	
	Median (P25, P75)	Median (P25, P75)	β coefficient (95%CI)	p value	β coefficient (95%CI)	p value
Radius 4% site						
Bone Mineral Content (g/cm)	1.655 (1.480, 1.800)	1.490 (1.430, 1.650)	− 0.170 (− 0.320, − 0.020)	0.026	− 0.197 (− 0.345, − 0.048)	0.009
Bone Total Area (mm^2^)	510.9 (455.8, 558.6)	460.5 (426.5, 511.5)	− 50.000 (− 107.960, 7.960)	0.091	− 44.326 (− 88.140, − 0.511)	0.047
Bone Total Density (mg/cm^3^)	331.0 (296.3, 360.7)	317.5 (290.1, 343.3)	− 13.390 (− 43.669, 16.889)	0.385	− 3.586 (− 29.881, 22.709)	0.789
Bone Trabecular Area (mm^2^)	229.8 (205.0, 251.3)	207.0 (191.8, 230.0)	− 22.500 (− 48.545, 3.545)	0.090	− 20.274 (− 40.002, − 0.546)	0.044
Bone Trabecular Density (mg/cm^3^)	217.2 (188.6, 242.7)	221.1 (187.6, 233.6)	3.820 (− 22.320, 29.960)	0.774	8.375 (− 15.821, 32.572)	0.496
Bone Cortical Area (mm^2^)	281.1 (250.8, 307.3)	253.5 (234.8, 281.5)	− 27.500 (− 59.415, 4.415)	0.091	− 24.052 (− 48.139, 0.035)	0.050
Bone Cortical Density (mg/cm^3^)	419.6 (375.7, 462.2)	391.6 (368.1, 433.0)	− 28.200 (− 69.567, 13.167)	0.181	− 14.442 (− 53.919, 25.036)	0.472
Radius 66% site						
Bone Mineral Content (g/cm)	1.400 (1.270, 1.520)	1.315 (1.220, 1.398)	− 0.090 (− 0.220, 0.040)	0.175	− 0.143 (− 0.266, − 0.020)	0.023
Bone Total Area (mm^2^)	176.0 (161.9, 194.8)	158.9 (146.2, 169.7)	− 13.000 (− 30.746, 4.746)	0.151	− 19.525 (− 33.489, − 5.561)	**0.006**
Bone Total Density (mg/cm^3^)	804.3 (732.7, 855.5)	844.7 (774.6, 885.2)	25.500 (− 42.002, 93.002)	0.458	32.065 (− 23.822, 87.953)	0.260
Bone Cortical Area (mm^2^)	108.5 (97.1, 117.6)	103.4 (93.4, 105.5)	− 6.500 (− 17.359, 4.359)	0.240	− 7.553 (− 17.579, 2.474)	0.139
Bone Cortical Density (mg/cm^3^)	1146.8 (1119.7, 1168.4)	1153.0 (1138.2, 1171.4)	5.330 (− 19.911, 30.571)	0.678	7.522 (− 11.445, 26.490)	0.436
Bone Cortical Thickness (mm)	2.846 (2.564, 3.084)	2.913 (2.663, 3.048)	0.056 (− 0.206, 0.318)	0.675	0.041 (− 0.184, 0.265)	0.721
Polar Stress Strain Index (mm^3^)	418.9 (363.7, 475.1)	367.7 (330.8, 416.9)	− 51.090 (− 109.422, 7.242)	0.086	− 46.500 (− 95.400, 2.399)	0.062
Tibia 4% site						
Bone Mineral Content (g/cm)	4.280 (3.860, 4.640)	4.095 (3.525, 4.603)	− 0.200 (− 0.609, 0.209)	0.336	− 0.429 (− 0.804, − 0.054)	0.025
Bone Total Area (mm^2^)	1325.8 (1218.8, 1431.8)	1296.9 (1238.4, 1451.1)	− 8.750 (− 107.337, 89.836)	0.862	− 37.702 (− 111.172, 35.768)	0.314
Bone Total Density (mg/cm^3^)	320.1 (298.4, 351.9)	318.1 (265.5, 331.5)	− 6.740 (− 32.175, 18.695)	0.603	− 8.662 (− 30.491, 13.167)	0.436
Bone Trabecular Area (mm^2^)	596.5 (548.3, 644.3)	583.5 (557.3, 652.9)	− 4.000 (− 48.243, 40.243)	0.859	− 25.366 (− 58.448, 7.717)	0.132
Bone Trabecular Density (mg/cm^3^)	254.2 (229.0, 276.1)	251.7 (226.5, 278.0)	− 3.240 (− 27.727, 21.247)	0.795	− 4.600 (− 28.845, 19.645)	0.709
Bone Cortical Area (mm^2^)	729.3 (670.5, 787.5)	713.4 (681.2, 798.3)	− 4.750 (− 59.093, 49.593)	0.864	− 30.916 (− 71.313, 9.482)	0.133
Bone Cortical Density (mg/cm^3^)	376.7 (350.2, 414.7)	350.1 (305.4, 389.7)	− 20.850 (− 47.613, 5.913)	0.126	− 15.820 (− 40.657, 9.017)	0.211
Tibia 66% site						
Bone Mineral Content (g/cm)	4.700 (4.300, 5.080)	4.515 (4.075, 5.105)	− 0.080 (− 0.484, 0.324)	0.697	− 0.255 (− 0.562, 0.051)	0.102
Bone Total Area (mm^2^)	754.8 (695.3, 821.0)	729.1 (658.6, 873.1)	− 19.750 (− 82.975, 43.475)	0.539	− 38.770 (− 95.476, 17.936)	0.180
Bone Total Density (mg/cm^3^)	624.8 (570.4, 679.3)	626.6 (575.8, 664.8)	− 8.970 (− 61.616, 43.676)	0.738	− 7.961 (− 58.615, 42.692)	0.757
Bone Cortical Area (mm^2^)	356.3 (320.9, 385.9)	336.6 (301.4, 381.7)	− 0.750 (− 31.267, 29.767)	0.961	− 20.081 (− 47.658, 7.496)	0.153
Bone Cortical Density (mg/cm^3^)	1112.7 (1087.6, 1133.5)	1115.8 (1091.2, 1137.2)	− 1.160 (− 22.823, 20.503)	0.916	4.445 (− 16.287, 25.177)	0.674
Bone Cortical Thickness (mm)	4.267 (3.805, 4.704)	4.058 (3.758, 4.582)	− 0.188 (− 0.617, 0.241)	0.389	− 0.090 (− 0.510, 0.330)	0.675
Polar Stress Strain Index (mm^3^)	3298.0 (2931.6, 3629.3)	3141.0 (2856.0, 3708.0)	− 138.240 (− 480.635, 204.156)	0.428	− 216.243 (− 446.390, 13.905)	0.065

Bold indicates a statistically significant difference

*Other variables tested in the models: age, weight, height, mobility, smoking status, alcohol consumption, prior fracture, Charlson comorbidity index, angiotensin-converting enzyme inhibitors, angiotensin II receptor blockers, diuretics and dihydropyridine calcium channel blockers

There were no differences observed between men with and without possible primary aldosteronism (ARR ≥ 70 pmol/mIU or taking a RAAS affecting medication except beta blocker and renin < 15 mU/L, n = 32). However, there was a trend towards lower adjusted bone mineral content at the radial and tibial 4% sites, as well as polar stress strain index at the tibial 66% site (Table 4).

**Table 4:** Relationship between peripheral quantitative computed tomography derived bone parameters and possible primary aldosteronism status: either (i) Aldosterone-renin-ratio (ARR) ≥ 70 pmol/mIU or (ii) taking a medication that affects the renin–angiotensin–aldosterone system, but not a beta blocker and renin < 15 mU/L

	Summary of bone measure by possible primary aldosteronism category		Comparison of bone measures between the two categories (reference = Unlikely)			
	Unlikely (n = 399)	Possible (n = 32)	Unadjusted		Adjusted*	
	Median (P25, P75)	Median (P25, P75)	β coefficient (95%CI)	p value	β coefficient (95%CI)	p value
Radius 4% site						
Bone Mineral Content (g/cm)	1.660 (1.490–1.800)	1.520 (1.430–1.680)	− 0.140 (− 0.249, − 0.031)	0.012	− 0.142 (− 0.255, − 0.029)	0.014
Bone Total Area (mm^2^)	508.4 (454.4–558.9)	502.3 (443.0–522.0)	− 6.500 (− 50.822, 37.822)	0.773	− 21.005 (− 54.191, 12.181)	0.214
Bone Total Density (mg/cm^3^)	331.4 (297.7–361.0)	311.3 (278.6–343.8)	− 19.870 (− 41.264, 1.524)	0.069	− 4.206 (− 23.768, 15.357)	0.673
Bone Trabecular Area (mm^2^)	228.6 (204.4–251.4)	226.0 (199.3–234.9)	− 2.750 (− 22.766, 17.266)	0.787	− 9.638 (− 24.650, 5.374)	0.208
Bone Trabecular Density (mg/cm^3^)	217.7 (189.6–243.0)	208.1 (179.1–233.1)	− 9.760 (− 29.017, 9.497)	0.320	− 7.090 (− 24.893, 10.713)	0.434
Bone Cortical Area (mm^2^)	279.8 (250.0–307.4)	276.3 (243.8–287.1)	− 3.750 (− 28.056, 20.556)	0.762	− 11.367 (− 29.560, 6.826)	0.220
Bone Cortical Density (mg/cm^3^)	419.9 (376.6–462.7)	396.0 (358.8–434.2)	− 24.010 (− 55.754, 7.734)	0.138	− 12.362 (− 40.187, 15.462)	0.383
Radius 66% site						
Bone Mineral Content (g/cm)	1.400 (1.270–1.520)	1.350 (1.225–1.453)	− 0.070 (− 0.165, 0.025)	0.146	− 0.075 (− 0.167, 0.017)	0.109
Bone Total Area (mm^2^)	175.6 (160.3–194.6)	171.3 (156.8–188.7)	− 4.500 (− 16.869, 7.869)	0.475	− 6.708 (− 16.772, 3.357)	0.191
Bone Total Density (mg/cm^3^)	808.4 (733.8–858.4)	801.8 (728.3–855.8)	− 7.550 (− 58.602, 43.502)	0.771	13.817 (− 28.652, 56.287)	0.523
Bone Cortical Area (mm^2^)	108.5 (97.3–117.8)	105.1 (93.3–110.3)	− 3.500 (− 11.312, 4.312)	0.379	− 5.136 (− 12.151, 1.878)	0.151
Bone Cortical Density (mg/cm^3^)	1147.9 (1120.3–1168.7)	1137.4 (1115.8–1157.8)	− 9.060 (− 26.503, 8.383)	0.308	1.551 (− 12.428, 15.529)	0.827
Bone Cortical Thickness (mm)	2.853 (2.571–3.086)	2.811 (2.476–3.029)	− 0.078 (− 0.269, 0.113)	0.423	0.023 (− 0.140, 0.185)	0.782
Polar Stress Strain Index (mm^3^)	418.9 (363.7–476.5)	381.5 (348.8–447.4)	− 34.770 (− 75.536, 5.996)	0.094	− 31.027 (− 65.664, 3.610)	0.079
Tibia 4% site						
Bone Mineral Content (g/cm)	4.280 (3.848–4.640)	4.140 (3.660–4.605)	− 0.140 (− 0.443, 0.163)	0.364	− 0.365 (− 0.642, − 0.088)	0.010
Bone Total Area (mm^2^)	1324.8 (1212.1–1431.4)	1325.3 (1239.6–1457.5)	− 0.500 (− 78.031, 77.031)	0.990	− 15.389 (− 73.356, 42.579)	0.602
Bone Total Density (mg/cm^3^)	320.3 (298.9–351.9)	306.2 (277.6–330.9)	− 14.120 (− 32.090, 3.850)	0.123	− 8.830 (− 25.620, 7.959)	0.302
Bone Trabecular Area (mm^2^)	596.0 (545.4–644.1)	596.3 (557.8–655.8)	− 0.250 (− 35.107, 34.607)	0.989	− 6.768 (− 32.910, 19.373)	0.611
Bone Trabecular Density (mg/cm^3^)	255.0 (229.2–276.2)	245.2 (225.3–276.0)	− 9.340 (− 27.864, 9.184)	0.322	− 10.940 (− 29.363, 7.483)	0.244
Bone Cortical Area (mm^2^)	728.8 (666.7–787.3)	729.0 (681.9–801.8)	− 0.250 (− 42.924, 42.424)	0.991	− 8.306 (− 40.198, 23.586)	0.609
Bone Cortical Density (mg/cm^3^)	377.6 (352.4–414.7)	352.8 (317.1–392.1)	− 24.950 (− 44.056, − 5.844)	0.011	− 12.266 (− 31.508, 6.977)	0.211
Tibia 66% site						
Bone Mineral Content (g/cm)	4.710 (4.305–5.090)	4.505 (4.130–4.825)	− 0.180 (− 0.497, 0.137)	0.265	− 0.257 (− 0.486, − 0.028)	0.028
Bone Total Area (mm^2^)	754.8 (693.5–821.3)	729.4 (700.1–820.4)	− 19.750 (− 68.073, 28.573)	0.422	− 41.576 (− 84.014, 0.861)	0.055
Bone Total Density (mg/cm^3^)	626.0 (570.4–681.9)	592.7 (572.6–657.5)	− 31.920 (− 70.229, 6.389)	0.102	− 11.294 (− 50.402, 27.815)	0.570
Bone Cortical Area (mm^2^)	356.8 (321.4–387.0)	344.4 (303.5–371.9)	− 9.750 (− 33.890, 14.390)	0.427	− 18.603 (− 38.023, 0.817)	0.060
Bone Cortical Density (mg/cm^3^)	1113.2 (1087.2–1133.6)	1108.9 (1092.2–1132.8)	− 5.320 (− 22.075, 11.436)	0.533	4.445 (− 11.469, 20.358)	0.583
Bone Cortical Thickness (mm)	4.272 (3.806–4.720)	4.014 (3.758–4.538)	− 0.244 (− 0.565, 0.077)	0.136	− 0.134 (− 0.460, 0.192)	0.418
Polar Stress Strain Index (mm^3^)	3300.8 (2931.6–3645.8)	3141.0 (2856.0–3551.0)	− 141.030 (− 396.277, 114.217)	0.278	− 219.655 (− 384.675, − 54.635)	0.009

The reference group (“unlikely” primary aldosteronism) included men that did not meet either criteria (i) or (ii)

*Other variables tested in the models: age, weight, height, mobility, smoking status, alcohol consumption, prior fracture, Charlson comorbidity index, angiotensin-converting enzyme inhibitors, angiotensin II receptor blockers, diuretics and dihydropyridine calcium channel blockers

There were no associations observed between aldosterone concentration as a continuous variable and any of the bone parameters derived from pQCT (Table 5).

**Table 5:** Associations between aldosterone concentration (pmol/L, natural log transformed) and peripheral quantitative computed tomography derived bone parameters

	Results from median regression for the association between aldosterone concentration (pmol/L) and bone parameters			
	Unadjusted		Adjusted^*^	
	β coefficient (95%CI)	p value	β coefficient (95%CI)	p value
Radius 4% site				
Bone Mineral Content (g/cm)	0.018 (− 0.051, 0.087)	0.615	0.033 (− 0.034, 0.100)	0.336
Bone Total Area (mm^2^)	− 9.423 (− 32.371, 13.525)	0.420	− 5.533 (− 26.052, 14.985)	0.596
Bone Total Density (mg/cm^3^)	10.707 (− 2.299, 23.714)	0.106	8.174 (− 3.272, 19.621)	0.161
Bone Trabecular Area (mm^2^)	− 4.107 (− 14.445, 6.230)	0.435	− 2.481 (− 11.695, 6.732)	0.597
Bone Trabecular Density (mg/cm^3^)	7.809 (− 4.100, 19.718)	0.198	8.369 (− 1.905, 18.644)	0.110
Bone Cortical Area (mm^2^)	− 5.316 (− 17.926, 7.295)	0.408	− 3.044 (− 14.309, 8.222)	0.596
Bone Cortical Density (mg/cm^3^)	19.594 (1.933, 37.255)	0.030	17.038 (1.308, 32.768)	0.034
Radius 66% site				
Bone Mineral Content (g/cm)	0.010 (− 0.048, 0.069)	0.729	− 0.025 (− 0.074, 0.024)	0.322
Bone Total Area (mm^2^)	− 4.405 (− 11.417, 2.607)	0.217	− 4.252 (− 9.911, 1.407)	0.140
Bone Total Density (mg/cm^3^)	27.009 (− 1.867, 55.884)	0.067	16.997 (− 7.325, 41.319)	0.170
Bone Cortical Area (mm^2^)	0.000 (− 4.564, 4.564)	> 0.999	− 0.694 (− 4.391, 3.003)	0.712
Bone Cortical Density (mg/cm^3^)	9.011 (− 1.344, 19.366)	0.088	6.406 (− 1.468, 14.281)	0.110
Bone Cortical Thickness (mm)	0.063 (− 0.034, 0.160)	0.200	0.052 (− 0.039, 0.143)	0.261
Polar Stress Strain Index (mm^3^)	− 3.143 (− 26.865, 20.579)	0.795	− 5.192 (− 24.658, 14.274)	0.600
Tibia 4% site				
Bone Mineral Content (g/cm)	0.134 (− 0.041, 0.309)	0.134	0.120 (− 0.041, 0.281)	0.143
Bone Total Area (mm^2^)	− 5.599 (− 50.905, 39.706)	0.808	1.193 (− 34.680, 37.066)	0.948
Bone Total Density (mg/cm^3^)	5.254 (− 5.436, 15.944)	0.334	2.578 (− 7.839, 12.994)	0.627
Bone Trabecular Area (mm^2^)	− 2.678 (− 23.070, 17.714)	0.796	0.693 (− 14.944, 16.330)	0.931
Bone Trabecular Density (mg/cm^3^)	2.006 (− 9.494, 13.507)	0.732	0.947 (− 10.545, 12.439)	0.871
Bone Cortical Area (mm^2^)	− 2.948 (− 27.907, 22.011)	0.816	0.518 (− 19.232, 20.268)	0.959
Bone Cortical Density (mg/cm^3^)	8.998 (− 2.489, 20.486)	0.124	9.347 (− 1.986, 20.679)	0.106
Tibia 66% site				
Bone Mineral Content (g/cm)	0.137 (− 0.046, 0.321)	0.143	− 0.006 (− 0.151, 0.139)	0.936
Bone Total Area (mm^2^)	− 2.287 (− 31.101, 26.528)	0.876	0.642 (− 24.734, 26.019)	0.960
Bone Total Density (mg/cm^3^)	− 1.976 (− 26.530, 22.577)	0.874	− 4.957 (− 27.449, 17.534)	0.665
Bone Cortical Area (mm^2^)	10.713 (− 3.599, 25.024)	0.142	5.928 (− 5.734, 17.591)	0.318
Bone Cortical Density (mg/cm^3^)	− 0.996 (− 11.525, 9.533)	0.852	− 0.978 (− 10.263, 8.307)	0.836
Bone Cortical Thickness (mm)	0.173 (− 0.033, 0.380)	0.100	0.113 (− 0.087, 0.313)	0.268
Polar Stress Strain Index (mm^3^)	− 15.667 (− 175.002, 143.668)	0.847	− 23.782 (− 127.070, 79.505)	0.651

*Other variables tested in the models: age, weight, height, mobility, smoking status, alcohol consumption, prior fracture, Charlson comorbidity index, angiotensin-converting enzyme inhibitors, angiotensin II receptor blockers, diuretics and dihydropyridine calcium channel blockers

### Impact Microindentation (IMI)

For IMI, 332 men completed the measurement. Exclusions for 99 men were: excessive soft tissue over the tibia (n = 65), skin condition (n = 14), needle phobia (n = 7), discomfort during measurement (pressure, not pain, n = 4), no reason given (n = 8) and unable to provide informed consent (n = 1).

The median BMSi among all men was 83.1 (P25, P75: 78.5 to 87.3). There was no association detected between ARR (continuous variable) and BMSi in unadjusted (β = 0.671 95%CI − 0.086, 1.428; p = 0.082) or adjusted analyses (β = − 0.029 95%CI − 0.756, 0.698; p = 0.938).

The median BMSi for men with likely primary aldosteronism (n = 10) was comparable to those with lower ARR (83.3 [P25, P75: 75.4–86.6] vs 83.1 [P25, P75: 78.5–87.3], p = 0.643). In adjusted analyses, no differences were observed (β = − 0.124 95%CI − 4.751, 4.503; p = 0.958).

The median BMSi for men with possible primary aldosteronism (ARR ≥ 70 pmol/mIU or taking a RAAS affecting medication except beta blocker and renin < 15 mU/L, n = 19) was also similar to those who did not meet the criteria (85.7 [P25, P75: 79.6–87.4] vs 83.0 [P25, P75: 78.5–87.3], p = 0.459). In adjusted analyses, no differences were observed (β = 1.054 95%CI − 2.300, 4.408; p = 0.537).

For aldosterone concentration (continuous variable), there was no association with BMSi in unadjusted (β = 1.008 95%CI − 0.828, 2.844; p = 0.281) or adjusted (β = 0.177 95%CI − 1.460, 1.813; p = 0.832) analyses.

For models (both pQCT and IMI) that included BMI and height instead of weight and height, the results were similar (data not shown). Excluding prior fracture also did not change the results.

## Discussion

This study investigated associations between ARR and bone parameters derived from pQCT and IMI techniques. The ARR as a continuous variable was not associated with any pQCT-derived bone parameters. Additionally, no associations were observed for aldosterone concentration as a continuous variable with any of the bone parameters. However, the men who had likely primary aldosteronism (ARR ≥ 70 pmol/mIU [3]) had lower bone total area (66% radial site) compared to those who did not have likely primary aldosteronism. There were also trends towards lower bone mineral content (4% radial and tibial, 66% radial sites). When we also included men who had low renin concentrations (< 15 mU/L) despite taking RAAS-active medications which should increase renin (those taking beta blockers were excluded), the results were similar, with trends towards lower bone mineral content (4% radial and tibial sites) and polar stress strain index (66% tibial site) in men who met these criteria. The association for lower adjusted bone area in men with likely primary aldosteronism was only observed following adjustment for other variables, in particular height, which was different between those with and without likely primary aldosteronism (Supplementary Table 1). Overall, the data may suggest that an ARR above the physiologic range could have deleterious effects on bone health.

The reason for the trends in bone deficits observed for men with likely/possible primary aldosteronism are not clear. However, compared to men without likely primary aldosteronism, those with likely primary aldosteronism had lower vitamin D. For the group of men meeting the definition for possible primary aldosteronism, there was a trend towards elevated parathyroid hormone and lower vitamin D. Similar observations with elevated parathyroid hormone and reduced vitamin D for individuals with primary aldosteronism have also been reported in other studies [6, 9, 10]. Additionally, a clinical trial [30] following men over an 8 year period, showed that for those with vitamin D deficiency (≤ 20 ng/mL), total BMD, cortical BMD, cortical area and cortical thickness measured using high-resolution pQCT at the distal radius all declined faster over time than for men with sufficient vitamin D. The study also reported that men in the two highest quartiles of parathyroid hormone had a faster decline in total BMD, cortical thickness, cortical area and cortical BMD at the distal radius than those in the lowest quartile of parathyroid hormone.

Another potential mechanism could be similar to that observed in primary hyperparathyroidism, characterised by chronically elevated parathyroid hormone concentrations, resulting in increased bone turnover, favouring bone resorption [31]. Elevated bone resorption can lead to bone loss and result in a lower bone mineral content. Men with possible primary aldosteronism also had higher levels of bone turnover markers, which supports this potential mechanism. Due to a lack of literature on this topic, we do not have a clear mechanism for the observed results, particularly for the changes in bone area. However, if these results are replicated in future work, novel research studies would be valuable to determine the mechanisms involved in associations between bone parameters and elevated ARR values.

Of note, IMI was not different between the groups. This might be explained either by the point of measurement, at the tibia, where the impact of the ARR seems less pronounced, or by an undetectable effect on the properties measured by the technique.

Only one previous study has examined associations between aldosterone, renin and ARR values with pQCT-derived bone parameters [16]. The study, which included 373 participants of African ancestry, reported that higher renin activity was associated with an increased trabecular BMD. These results are similar to our study, since higher renin activity corresponds to a lower ARR and there was a trend towards greater ARR and lower bone mineral content (tibia 4% site). The authors of the previous study suggested that since their participants were not selected on the basis of disease, BMD may not be affected unless the RAAS has been disrupted substantially. The ARR as a continuous variable may not have been associated with any of the pQCT bone parameters since the participants in our study were not selected on the basis of disease and most had ARR within the physiological range. Indeed, the analysis including men with and without likely primary aldosteronism showed that outside the normal physiological range of ARR, bone area was lower.

There are few studies investigating associations between ARR or primary aldosteronism and bone health. Individuals with primary aldosteronism have been reported to have a higher risk of fracture [9, 12] and poorer trabecular bone score [14]. Further studies are needed to confirm these observations, conduct similar analyses in women as well as explore the possible mechanisms for the bone health deficits for individuals with primary aldosteronism.

In cases where aldosterone concentrations are outside the normal range, treatments targeting these elevated concentrations may also result in improved bone health. In a review article describing primary aldosteronism and bone metabolism [9], the authors highlighted that bone loss in primary aldosteronism can be reversed with treatment (either medication or surgery). Additionally, a study by Hu et al. showed that anti-osteoporosis therapy was associated with altered concentrations of aldosterone and renin [32]. The study included postmenopausal women with osteoporosis, who were treated with either alendronate or parathyroid hormone for 48 weeks. The results showed that aldosterone and renin decreased after the treatment period. The authors also highlighted that women with postmenopausal osteoporosis can often have false-positive ARR screening and care should be taken when interpreting ARR levels. Zhang et al. [33] have described how treatment with a renin inhibitor resulted in increased trabecular bone in ovariectomised mice. Further investigation of the effects of excess aldosterone on bone may improve the understanding of osteopenia or osteoporosis that cannot be explained by another known cause [6].

This study has a number of strengths and limitations. One strength is that the participants were randomly selected from the population and the results are thus more generalisable than if the participants were selected on the basis of disease. An advantage of using a population-based sample was also a large sample size. However, there may be a bias towards healthier individuals participating in the study, which may have affected the results. The measurement of aldosterone and renin (and thus the calculated ARR values) may have been affected by several factors that we were not able to control, including posture, time of day, salt intake and medication [3]. Some of these factors were managed well in this study; for example, participants were asked to fast prior to providing a blood sample and thus 90% had their blood withdrawn in the morning, likely in a seated position. We did not ask participants to change their diet and thus salt intake was not controlled. Urinary sodium values as a marker of salt intake were not available, nor were urinary calcium values. Additionally, participants were not asked to modify their medications, but data for several important medication types were included in the analyses (ACEIs, ARBs, diuretics, dihydropyridine calcium channel blockers, beta blockers). Another limitation is that only one measurement of aldosterone and renin was performed and the number of participants with likely primary aldosteronism was small (N = 16). We used the criterion of ARR ≥ 70 pmol/mIU to categorise “likely” primary aldosteronism, however, it has been reported that ARR in the range 70–100 pmol/mIU is a “grey zone” [3] and that a value ≥ 100 pmol/mIU is a more robust cut-point. Unfortunately in this study we did not have a sufficient number of participants with ARR ≥ 100 pmol/mIU to use this higher cut-point. However, we also performed analyses with a larger group of men who had possible primary aldosteronism, specifically including those with a low renin concentration despite using medications which should increase renin, while excluding those taking beta blockers which tend to lower renin and increase the ARR. Although we included a range of potential confounders in the adjusted models, it is possible that there was residual confounding. The study included only men, which is important because there is likely a sex difference in the effect of aldosterone and renin on bone [34], and future studies will need to include female participants. At the time of writing, data are still being collected for pQCT and IMI in the next follow-up phase for women enrolled in the Geelong Osteoporosis Study (2022 onwards). Since this is a cross-sectional study, the effect on longitudinal outcomes such as fracture risk is unknown and would be an important research question for future studies.

## Conclusion

There were no associations detected between ARR or aldosterone concentration and bone parameters derived from pQCT. However, men with likely primary aldosteronism (ARR ≥ 70 pmol/mIU) had lower total bone area. The observations suggest that bone may be adversely affected when the ARR is above physiological levels. Future work should include women and longitudinal studies with larger sample size to determine the risk of fracture for individuals with elevated ARR.

### Supplementary Information

Below is the link to the electronic supplementary material.

Supplementary file1 (DOCX 23 KB)
