# Supplementary material for: Associations Between Aldosterone-Renin-Ratio and Bone Parameters Derived from Peripheral Quantitative Computed Tomography and Impact Microindentation in Men
Source: Calcif Tissue Int. 2023 Sep 10;113(5):496–510. doi: 10.1007/s00223-023-01131-x (PMC10618308; doi:10.1007/s00223-023-01131-x)
Supplement: Supplementary file 1 — Supplementary file1 (DOCX 23 KB) [file 223_2023_1131_MOESM1_ESM.docx]

Supplementary Table 1: Descriptive characteristics of the participants included in this study (N=431), stratified by likely primary aldosteronism status (Aldosterone-renin-ratio (ARR) ≥70 pmol/mIU). Data presented as mean±SD, median(P25, P75) or n (%), as appropriate.

|  | **ARR<70 (n=415)** | **ARR≥70 (n=16)** | **p value** |
| --- | --- | --- | --- |
| Age (years) (median P25, P75) | 64.7 (54.2-73.6) | 65.6 (56.0-72.5) | 0.815 |
| Weight (kg) (mean±SD) | 84.5±13.6 | 85.5±13.4 | 0.776 |
| Height (cm) (mean±SD) | 174.4±6.9 | 177.2±4.8 | **0.035** |
| Body mass index (kg/m^2^) (mean±SD) | 27.8±3.9 | 27.2±4.2 | 0.637 |
| Systolic blood pressure (mmHg) (mean±SD) | 140.4±16.5 | 146.3±17.6 | 0.201 |
| Diastolic blood pressure (mmHg) (mean±SD) | 79.0±9.7 | 84.8±12.4 | 0.084 |
| Hypertension (n %) | 42 (10.1) | 6 (37.5) | **<0.001** |
| Low mobility (n %) | 100 (24.1) | 0 (0.0) | **-** |
| Current smoker (n %) | 28 (6.8) | 1 (6.3) | - |
| High alcohol consumption^a^ (n %) | 88 (21.2) | 2 (12.5) | - |
| Prior fracture (n %) | 47 (11.3) | 2 (12.5) | - |
| Charlson comorbidity index (median P25, P75) | 0 (0-1) | 0 (0-1) | 0.952 |
| Socioeconomic status^b^ (n %) |  |  | 0.162 |
| Quintile 1 (most disadvantaged) | 71 (17.1) | 0 (0.0) |  |
| Quintile 2 | 77 (18.6) | 3 (18.8) |  |
| Quintile 3 | 98 (23.6) | 3 (18.8) |  |
| Quintile 4 | 107 (25.8) | 5 (31.3) |  |
| Quintile 5 (most advantaged) | 55 (13.3) | 5 (31.3) |  |
| Biochemical data |  |  |  |
| Aldosterone (pmol/L) (median P25, P75) | 277.0 (197.0-387.0) | 341.0 (277.5-415.3) | 0.085 |
| Renin (mIU/L) (median P25, P75) | 26.1 (14.8-59.8) | 3.0 (2.2-4.0) | **<0.001** |
| Aldosterone-Renin Ratio (pmol/mIU) (median P25, P75) | 10.8 (4.9-19.2) | 93.0 (82.4-149.2) | **<0.001** |
| Estimated glomerular filtration rate (mL/min/1.73m^2^) (median P25, P75) | 74.5 (65.0-85.0) | 75.5 (66.0-84.0) | 0.800 |
| Serum calcium (mmol/L) (mean±SD) | 2.25±0.09 | 2.24±0.09 | 0.454 |
| Parathyroid hormone (pmol/L) (median P25, P75) | 5.4 (4.3-7.0) | 6.4 (4.5-6.9) | 0.409 |
| Vitamin D (nmol/L) (median P25, P75) | 64.0 (48.0-78.0) | 52.0 (37.0-65.8) | **0.032** |
| Sodium (mmol/L) (median P25, P75) | 140 (139-142) | 140 (138-143) | 0.863 |
| Potassium (mmol/L) (median P25, P75) | 4.2 (4.1-4.5) | 4.2 (4.1-4.4) | 0.753 |
| C-terminal telopeptide (CTx, ng/L) (median P25, P75) | 361 (270-462) | 361 (327-614) | 0.179 |
| Procollagen type 1 N propeptide (P1NP, mcg/L) (median P25, P75) | 47 (37-58) | 50 (41-67) | 0.398 |
| Medication use |  |  |  |
| Angiotensin-converting enzyme inhibitors (n %) | 73 (17.6) | 1 (6.3) | - |
| Angiotensin II receptor blockers (n %) | 75 (18.1) | 0 (0.0) | - |
| Diuretics (n %) | 44 (10.6) | 0 (0.0) | - |
| Dihydropyridine calcium channel blockers (n %) | 49 (11.8) | 3 (18.8) | - |
| Calcium supplements (n %) | 20 (4.8) | 0 (0.0) | - |
| Beta blockers (n %) | 38 (9.2) | 3 (18.8) | - |

Missing data: IRSAD n=7, estimated glomerular filtration rate n=3, calcium level n=3, parathyroid hormone n=12, vitamin D level n=7, sodium level n=3, potassium level n=3, CTx n=4, P1NP n=4.

^a^High consumption defined as ≥30g alcohol per day.

^b^Index of Relative Socio-economic Advantage and Disadvantage.

Supplementary Table 2: Descriptive characteristics of the participants included in this study (N=431), stratified by possible primary aldosteronism status: either i) Aldosterone-renin-ratio (ARR) ≥70 pmol/mIU or ii) taking a medication that affects the renin-angiotensin-aldosterone system, but not a beta blocker and renin <15mU/L. The referent group (“unlikely” primary aldosteronism) included men that did not meet either criteria i) or ii). Data presented as mean±SD, median(P25, P75) or n (%), as appropriate.

|  | **Unlikely**  **(n=399)** | **Possible**  **(n=32)** | **p value** |
| --- | --- | --- | --- |
| Age (years) (median P25, P75) | 64.4 (53.7-73.5) | 69.1 (61.1-81.9) | **0.014** |
| Weight (kg) (mean±SD) | 84.4±13.6 | 85.4±13.1 | 0.683 |
| Height (cm) (mean±SD) | 174.4±7.0 | 175.0±5.9 | 0.594 |
| Body mass index (kg/m^2^) (mean±SD) | 27.7±3.9 | 27.9±4.2 | 0.793 |
| Systolic blood pressure (mmHg) (mean±SD) | 139.9±16.4 | 148.8±16.6 | **0.006** |
| Diastolic blood pressure (mmHg) (mean±SD) | 79.0±9.7 | 82.1±11.1 | 0.127 |
| Hypertension (n %) | 41 (10.3) | 7 (21.9) | **0.045** |
| Low mobility (n %) | 95 (23.8) | 5 (15.6) | 0.291 |
| Current smoker (n %) | 28 (7.0) | 1 (3.1) | - |
| High alcohol consumption^a^ (n %) | 87 (21.8) | 3 (9.4) | 0.096 |
| Prior fracture (n %) | 44 (11.0) | 5 (15.6) | 0.431 |
| Charlson comorbidity index (median P25, P75) | 0 (0-1) | 0 (0-1) | 0.827 |
| Socioeconomic status^b^ (n %) |  |  | 0.413 |
| Quintile 1 (most disadvantaged) | 69 (17.3) | 2 (6.3) |  |
| Quintile 2 | 71 (17.8) | 9 (28.1) |  |
| Quintile 3 | 93 (23.3) | 8 (25.0) |  |
| Quintile 4 | 104 (26.1) | 8 (25.0) |  |
| Quintile 5 (most advantaged) | 55 (13.8) | 5 (15.6) |  |
| Biochemical data |  |  |  |
| Aldosterone (pmol/L) (median P25, P75) | 277.0 (200.0-388.0) | 290.5 (191.8-367.3) | 0.927 |
| Renin (mIU/L) (median P25, P75) | 27.6 (16.0-61.8) | 6.0 (3.0-10.3) | **<0.001** |
| Aldosterone-Renin Ratio (pmol/mIU) (median P25, P75) | 10.6 (4.8-18.7) | 61.6 (24.1-94.4) | **<0.001** |
| Estimated glomerular filtration rate (mL/min/1.73m^2^) (median P25, P75) | 75.0 (65.0-85.0) | 73.5 (65.3-82.8) | 0.533 |
| Serum calcium (mmol/L) (mean±SD) | 2.26±0.09 | 2.24±0.09 | 0.245 |
| Parathyroid hormone (pmol/L) (median P25, P75) | 5.4 (4.3-6.9) | 6.2 (4.9-7.9) | **0.073** |
| Vitamin D (nmol/L) (median P25, P75) | 64.0 (48.0-78.0) | 53.5 (41.0-71.5) | **0.057** |
| Sodium (mmol/L) (median P25, P75) | 140 (139-141) | 141 (140-143) | **0.016** |
| Potassium (mmol/L) (median P25, P75) | 4.2 (4.1-4.5) | 4.2 (3.9-4.4) | 0.080 |
| C-terminal telopeptide (CTx, ng/L) (median P25, P75) | 357 (269-459) | 426 (338-626) | **0.007** |
| Procollagen type 1 N propeptide (P1NP, mcg/L) (median P25, P75) | 46 (37-58) | 54 (44-67) | **0.032** |
| Medication use |  |  |  |
| Angiotensin-converting enzyme inhibitors (n %) | 70 (17.5) | 4 (12.5) | - |
| Angiotensin II receptor blockers (n %) | 67 (16.8) | 8 (25.0) | - |
| Diuretics (n %) | 41 (10.3) | 3 (9.4) | - |
| Dihydropyridine calcium channel blockers (n %) | 43 (10.8) | 9 (28.1) | **-** |
| Calcium supplements (n %) | 20 (5.0) | 0 (0.0) | - |
| Beta blockers (n %) | 38 (6.5) | 3 (9.4) | - |

Missing data: IRSAD n=7, estimated glomerular filtration rate n=3, calcium level n=3, parathyroid hormone n=12, vitamin D level n=7, sodium level n=3, potassium level n=3, CTx n=4, P1NP n=4.

^a^High consumption defined as ≥30g alcohol per day.

^b^Index of Relative Socio-economic Advantage and Disadvantage.
